# Comprehensive analysis of necroptosis-related lncRNA signature with potential implications in tumor heterogeneity and prediction of prognosis in clear cell renal cell carcinoma

**DOI:** 10.1186/s40001-023-01194-4

**Published:** 2023-07-14

**Authors:** Hang Lin, Lingzhi Qu, Guanqiu Chen, Chunfang Zhang, Liqing Lu, Yongheng Chen

**Affiliations:** 1 Department of Oncology, NHC Key Laboratory of Cancer Proteomics, State Local Joint Engineering Laboratory for Anticancer Drugs, Xiangya Hospital, Central South University, Changsha, China; 2 Department of Urology, The First Affiliated Hospital of Xi’an Jiaotong University, Xi’an, China; 3 Department of Thoracic Surgery, Xiangya Hospital, Central South University, Changsha, China

**Keywords:** Necroptosis-related lncRNA, Clear cell renal cell carcinoma, Tumor heterogeneity, Prognosis

## Abstract

**Background:**

Necroptosis has been reported to play a critical role in occurrence and progression of cancer. The dysregulation of long non-coding RNAs (lncRNAs) is associated with the progression and metastasis of clear cell renal cell carcinoma (CCRCC). However, research on necroptosis-related lncRNAs in the tumor heterogeneity and prognosis of CCRCC is not completely unclear. This study aimed to analysis the tumor heterogeneity among CCRCC subgroups and construct a CCRCC prognostic signature based on necroptosis-related lncRNAs.

**Methods:**

Weighted gene co-expression network analysis (WGCNA) was performed to identify necroptosis-related lncRNAs. A preliminary classification of molecular subgroups was performed by non-negative matrix factorization (NMF) consensus clustering analysis. Comprehensive analyses, including fraction genome altered (FGA), tumor mutational burden (TMB), DNA methylation alterations, copy number variations (CNVs), and single nucleotide polymorphisms (SNPs), were performed to explore the potential factors for tumor heterogeneity among the three subgroups. Subsequently, we constructed a predictive signature by multivariate Cox regression. Nomogram, calibration curves, decision curve analysis (DCA), and time-dependent receiver-operating characteristics (ROC) were used to validate and evaluate the signature. Finally, immune correlation analyses, including immune-related signaling pathways, immune cell infiltration status and immune checkpoint gene expression level, were also performed.

**Results:**

Seven necroptosis-related lncRNAs were screened out by WGCNA, and three subgroups were classified by NMF consensus clustering analysis. There were significant differences in survival prognosis, clinicopathological characteristics, enrichments of immune-related signaling pathway, degree of immune cell infiltration, and expression of immune checkpoint genes in the various subgroups. Most importantly, we found that 26 differentially expressed genes (DEGs) among the 3 subgroups were not affected by DNA methylation alterations, CNVs and SNPs. On the contrary, these DEGs were associated with the seven necroptosis-related lncRNAs. Subsequently, the identified RP11-133F8.2 and RP11-283G6.4 by multivariate Cox regression analysis were involved in the risk model, which could serve as an independent prognostic factor for CCRCC. Finally, qRT-PCR confirmed the differential expression of the two lncRNAs.

**Conclusions:**

These findings contributed to understanding the function of necroptosis-related lncRNAs in CCRCC and provided new insights of prognostic evaluation and optimal therapeutic strategy for CCRCC.

**Supplementary Information:**

The online version contains supplementary material available at 10.1186/s40001-023-01194-4.

## Introduction

Kidney cancer is one of the most common malignant tumors in the genitourinary system, accounting for approximately 4.1% of all new cancer case in the United State in 2022 [1, 2]. Renal cell carcinoma (RCC), as the major form of kidney cancer, makes up approximately 90% of all renal malignancies [3]. Of these, clear cell renal cell carcinoma (CCRCC) is the most aggressive subtype representing approximately 70% of all RCCs [4]. Patients with early-stage localized primary RCC may be cured after surgical resection, but approximately 20–30% of patients eventually develop recurrent and metastatic RCC [5]. At present, a combination of immune checkpoint inhibitors or a combination of immune checkpoint inhibitor and tyrosine kinase inhibitors is the mainstay of treatment for patients with metastatic RCC [6]. In the last decade, due to the development of new tyrosine kinase inhibitors and immune checkpoint inhibitors, impressive advancements have been made in the treatment of RCC patients [7]. However, due to the lack of specific prognostic biomarkers, individual differences in drug sensitivity and obvious side effects of drugs, advanced RCC has a high mortality rate and a low 5-year survival rate [8]. Therefore, it is of vital importance to further explore a specific biomarker for the diagnosis and prognosis of CCRCC.

Necroptosis is a novel programmed form of necrotic cell death in a caspase-independent manner, which is mainly mediated by receptor-interacting protein kinase 1 (RIPK1), receptor-interacting protein kinase 3 (RIPK3), and mixed lineage kinase domain-like pseudokinase (MLKL) [9]. When activated RIPK1 interacts with RIPK3, a crucial complex, necrosome, is formed. In the necrosome, RIPK3 phosphorylates its substrate MLKL, which is then oligomerized and translocated to plasma membrane, ultimately leading to the execution of necroptosis [9, 10]. Necroptosis shares similar morphological features with necrosis, mainly characterized by the rupture of the cellular membrane, progressively translucent cytoplasm and swelling of organelles [9, 11]. When the cellular membrane is ruptured in necroptotic cells, the released cell contents can cause the exposure of damage-associated molecular patterns (DAMPs) and trigger strong inflammatory responses [9, 12]. Necroptosis has been reported to play a critical role not only in viral infection and development but also in the regulation of cancer biology, mainly manifested in tumorigenesis, cancer metastasis, cancer immunity and cancer subtypes [9, 13]. It is worth noting that necroptosis has the dual effects of promoting and reducing tumor growth depending on the tumor type and conditions [9]. In the different types of tumor cells, the expression of several key factors in necroptotic signaling pathways were decreased, which was associated with poor prognosis as well as promotion of tumor progression and metastasis [14–18]. At present, targeting necroptosis has become a novel cancer therapy for bypassing apoptosis-resistance and supporting antitumor immunity [9].

Long non-coding RNAs (lncRNAs) are a class of transcribed non-coding RNAs with a length of more than 200 nucleosides, which are widely distributed in the cytoplasm and nucleus [19]. Accumulating evidence has shown that lncRNAs are involved in the development and progression of cancer and implicated in various biological processes, such as cell proliferation, cell cycle, cell differentiation and apoptosis [20, 21]. Recent research has indicated that the dysregulation of lncRNAs is associated with the progression and metastasis of CCRCC [22–25]. However, research on necroptosis-related lncRNAs in the tumor heterogeneity and prognosis of CCRCC has not been reported, and the role of necroptosis-related lncRNAs in CCRCC remained unclear.

In this study, we analyzed the tumor heterogeneity among CCRCC subgroups and constructed a novel predictive signature based on necroptosis-related lncRNAs, aiming to explore the potential factors for tumor heterogeneity and its prognostic value in CCRCC patients.

## Materials and methods

### Data extraction

All transcriptome RNA-seq and genomics data and clinical characteristics of enrolled samples were downloaded from The Cancer Genome Atlas (TCGA) (https://portal.gdc.cancer.gov/). A total of 531 CCRCC samples were enrolled in this study, which 76 normal tissues extracted from the TCGA database were termed as the control group.

### Weighted gene co-expression network analysis

Weighted gene co-expression network analysis (WGCNA), a systematic biological method, is used to construct a gene co-expression network to explore the relationship between network modules and clinical traits [26]. In this study, the expression profiles of lncRNAs in CCRCC samples enrolled samples was applied to construct gene co-expression networks using the “WGCNA” package in R software [26]. The construction process was the same as described previously [26, 27].

### Non-negative matrix factorization clustering analysis

Non-negative matrix factorization (NMF) is an efficient method whose algorithm divides the original matrix into two non-negative matrices to identify the potential feature in the gene expression profile [28]. In this study, we first integrated survival information of TCGA-KIRC and gained the necroptosis-related lncRNAs by WGCNA. Second, non-negative matrix factorization clustering was applied for identification of new subtypes using the “NMF” package in R software. All the necroptosis-related lncRNAs were selected to construct a principal component analysis (PCA) scoring system with the “prcomp” function in R software. The difference in survival probability among these subgroups was analyzed using the “survival” package in R software by Kaplan–Meier analysis and log-rank test. Third, the differential analysis of tumor clinicopathological characteristics among these subgroups was performed using the cBioportal, and the difference in fraction genome altered (FGA) and tumor mutational burden (TMB) were also analyzed using the cBioportal at the same time. Then, DNA methylation alterations, copy number variations (CNVs) and single nucleotide polymorphisms (SNPs) were further analyzed using the cBioportal and the “maftools” package in R software.

### Tumor immune analysis

Gene set enrichment analyses (GSEA) was performed to identify significantly enriched immune-related signaling pathways using the “clusterprofiler” package in R software [29]. Values of *p* < 0.05 and FDR < 0.25 were defined as thresholds for statistical significance. The differential expression of tumor-infiltrating immune cells (TIICs) among subgroups was evaluated using CIBERSORT algorithm. In addition, we also compare to the expression level of immune checkpoint gene among subgroups. The degree of difference was noted: * if *p* < 0.05, ** if* p* < 0.01, and *** if *p* < 0.001.

### Establishment of the risk model of CCRCC

The prognostic necroptosis-related lncRNAs screened were used for multivariate Cox regression analysis and risk model construction. The risk score was calculated using following algorithm: Risk Score = ${\sum }_{i=1}^{n} expression\, value \left(lncRNA i\right)\times\, regression \,coefficient(lncRNA i)$.

### Construction and calibration of predictive nomogram

A nomogram was created to predict the 1-, 3-, and 5-year OS of CCRCC patients based on multivariate Cox regression. Subsequently, a calibration curve was used to illustrate the predictive power the established nomogram model. Decision curve analysis (DCA) was used to compare the clinical benefits conferred by the prognostic evaluation of the nomogram and a single predictor. Receiver operator characteristic (ROC) curves were established to evaluate the diagnostic ability of nomogram and individual predictors.

### Quantitative real-time polymerase chain reaction (qRT-PCR)

HK-2 (human renal proximal convoluted tubule cell line) and 786-O (CCRCC cell line) are obtained from National Collection of Authenticated Cell Cultures, China. Total RNA was extracted with TRIzol reagent (Beyotime, China). Total RNA was reversely transcripted into cDNA. qPCR amplification was performed using the SYBR Green PCR kit (Servicebio, China) according to the manufacturer’s protocol. The PCR parameters were set for an initial cycle of 1 min at 95 °C, followed by a total of 40 cycles at 95 °C for 20 s, 55 °C for 20 s, and 72 °C for 30 s. The relative expression of each gene was normalized to human GAPDH levels and calculated using the 2^−ΔΔCt^ method. Experiments were repeated three times. The primer sequences for PCR amplification were as follows:

RP11-133F8.2, forward: 5′-CGAAGCCAAGCAAAGCAACA-3′,

Reverse: 5′-TCGCCCAAACACTTAAACGC-3′;

RP11-283G6.4, forward: 5′-AGTTGGAACTTGTGACCAGCA-3′,

Reverse: 5′-AGCCTCACTTTGGCAGGAAC-3′.

### Statistical analysis

The statistical analyses were performed with R Studio software (version 1.3.1093; https://rstudio.com/products/rstudio/). Statistical significance levels were determined by two-sided tests and *p* < 0.05 was considered statistically significant. The Mann–Whitney *U* test and *t*-test were used for continuous variables analysis and the χ^2^ test for categorical variables analysis. Univariate and multivariate Cox proportional hazard regression analyses were used for determining the risk model of necroptosis-related lncRNAs. The Kaplan–Meier method with a two-sided log-rank test was used for survival analysis.

## Results

### Identification of differentially expressed lncRNAs

**Fig. 1:**
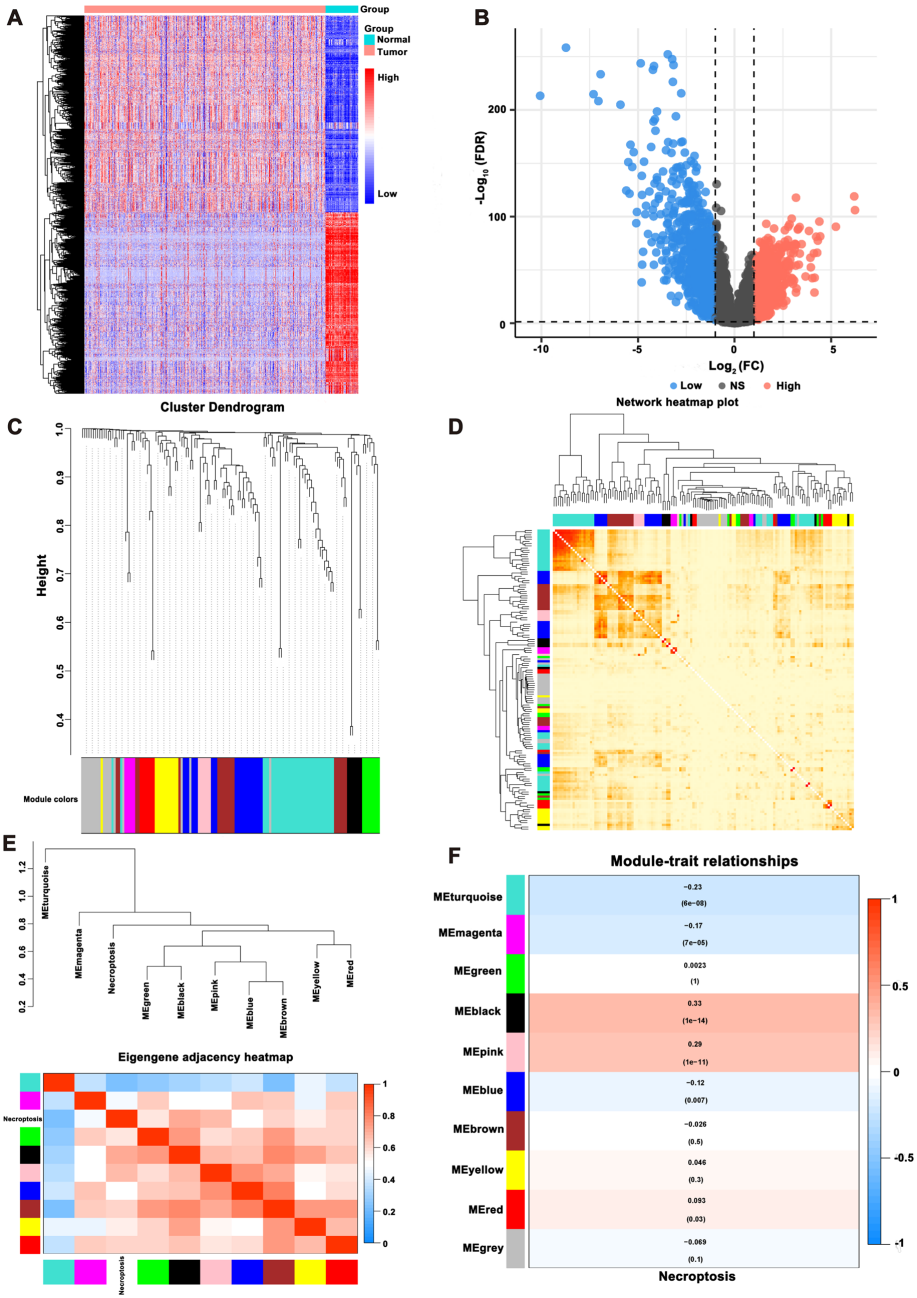
Identification of necroptosis-related lncRNAs in CCRCC. **A** The heatmap of differentially expressed genes. **B** The volcano plot of the differentially expressed genes. **C** The cluster dendrogram of co-expression network modules. **D** The heatmap of topological overlap in the gene network. **E** The heatmap of eigengene adjacency. **F** The module–trait relationships

We collected RNA-seq data of enrolled samples from 531 CCRCC patients and 76 normal tissues from TCGA. These data were used for differential expression analysis, and 138 lncRNAs were significantly differentially expressed between CCRCC and normal groups (Additional file 3: Table S1). The expression profiles of differentially expressed genes (DEGs) were visualized in the form of heat map and volcano map (Fig. 1A and B, respectively). Then, we obtained 67 necroptosis-related genes according to previously reported literature (Additional file 4: Table S2) [30, 31].

### Construction of co-expression network in CCRCC

WGCNA was performed to analyze the expression values of 138 lncRNAs in 531 enrolled samples. The soft-thresholding power was six which was determined according to a scale-free topology fit index (*R*^2^ = 0.85) (Additional file 1: Fig. S1). Therefore, the network conformed to the power-law distribution and was closer to the real biological network state. Resulting gene clustering tree and respective module colors are shown in Fig. 1C. The number of lncRNAs per module was noted in Additional file 5: Table S3. The topological overlap in the gene network is revealed in Fig. 1D. The heat map indicated the eigengene adjacency of modules (Fig. 1E). Our study focusses on the mechanism of necroptosis-related lncRNAs in CCRCC. Therefore, we mainly focused the black module (*r* = 0.33, *p* = 1e−14), which had the strongest correlation with the clinical characteristics, biologically (Fig. 1D). Finally, seven necroptosis-related lncRNAs, including TTC21B-AS1, RP4-764O22.1, RP11-133F8.2, RP11-283G6.4, AC073115.6, AC073115.7, and LINC01428 (Additional file 5: Table S3), were screened out for the subsequent analysis.

### NMF clustering of necroptosis-related lncRNAs in CCRCC

**Fig. 2:**
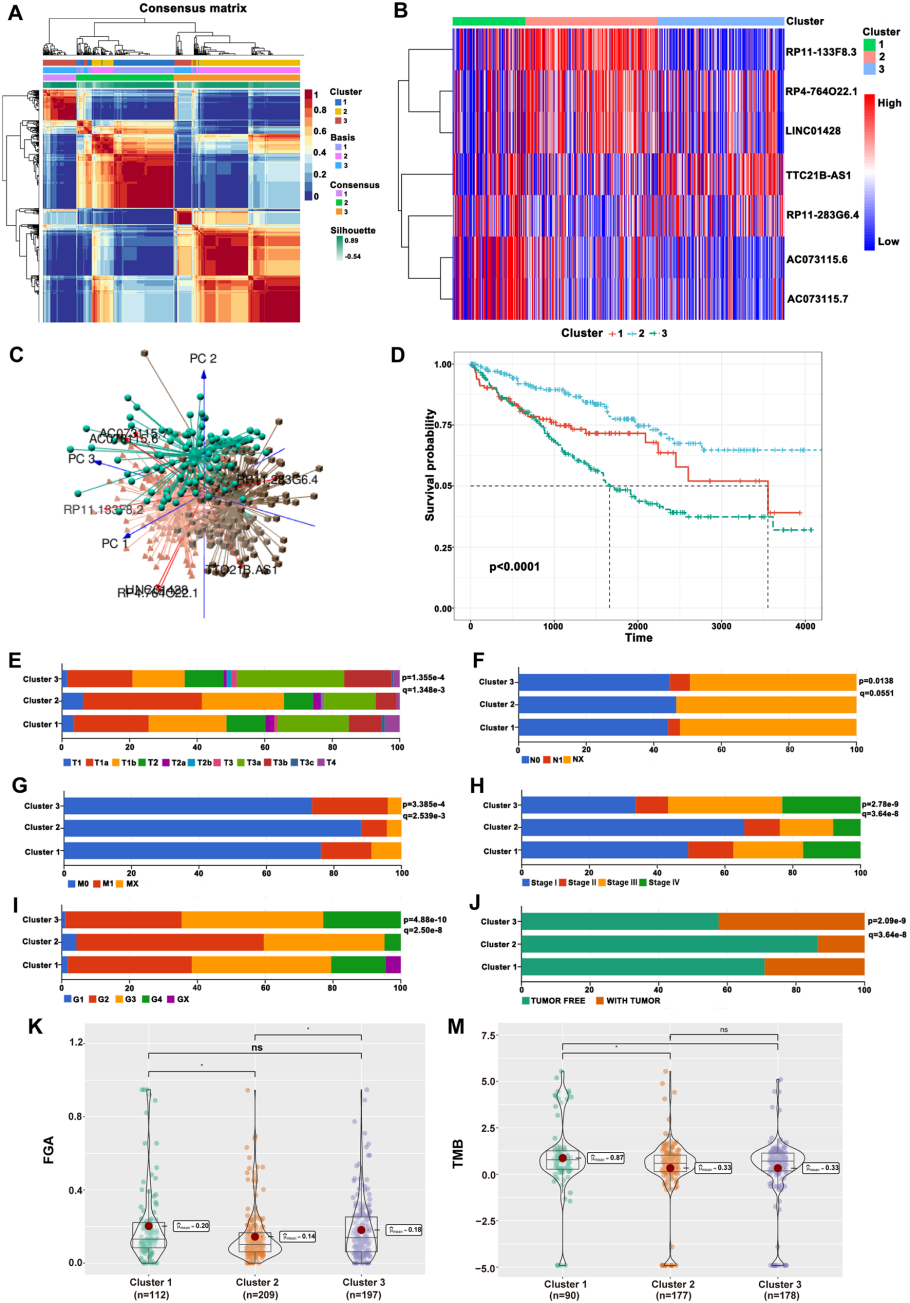
Tumor classification based on the identified seven necroptosis-related lncRNAs. **A** Three subgroups were classified by NMF consensus clustering analysis (*k* = 3). **B** The heatmap of seven necroptosis-related lncRNAs in the three subgroups. **C** The PCA of seven necroptosis-related lncRNAs for the three subgroups. **D** Kaplan–Meier survival curves in the three subgroups. **E**–**J** The comparison of differential clinicopathological characteristics in the three subgroups. **K** The difference of FDA in the three subgroups. **M** The difference of TMB in the three subgroups

Based on the expression of these identified necroptosis-related lncRNAs, a preliminary classification of molecular subgroups was performed by NMF consensus clustering analysis, and three subgroups were reasonably classified (Fig. 2A). As shown in Fig. 2B, the expression profiles of seven necroptosis-related lncRNAs in the three subgroups were visualized using a heatmap. PCA was applied to further verification the distinction among the three subgroups at the expression patterns of seven necroptosis-related lncRNAs (Fig. 2C). Kaplan–Meier survival curves indicated significantly differences in survival among the three subgroups (*p* < 0.0001) (Additional file 6: Table S4), and Cluster 2 had a better survival probability than Cluster 1 and Cluster 3 (Fig. 2D). The clinicopathological characteristics of the three subgroups are presented in Fig. 2E–J, which showed that the differences in T stage, M stage, pathologic stage, histologic grade and tumor status were statistically significant. These results suggest tumor heterogeneity among the three subgroups. In addition, FGA and TMB had obvious difference between subgroups (Fig. 2K–M), which prompted us to further analyze the effect of these alterations on mRNA expression levels.

## Analysis of tumor heterogeneity among CCRCC subgroups

**Fig. 3:**
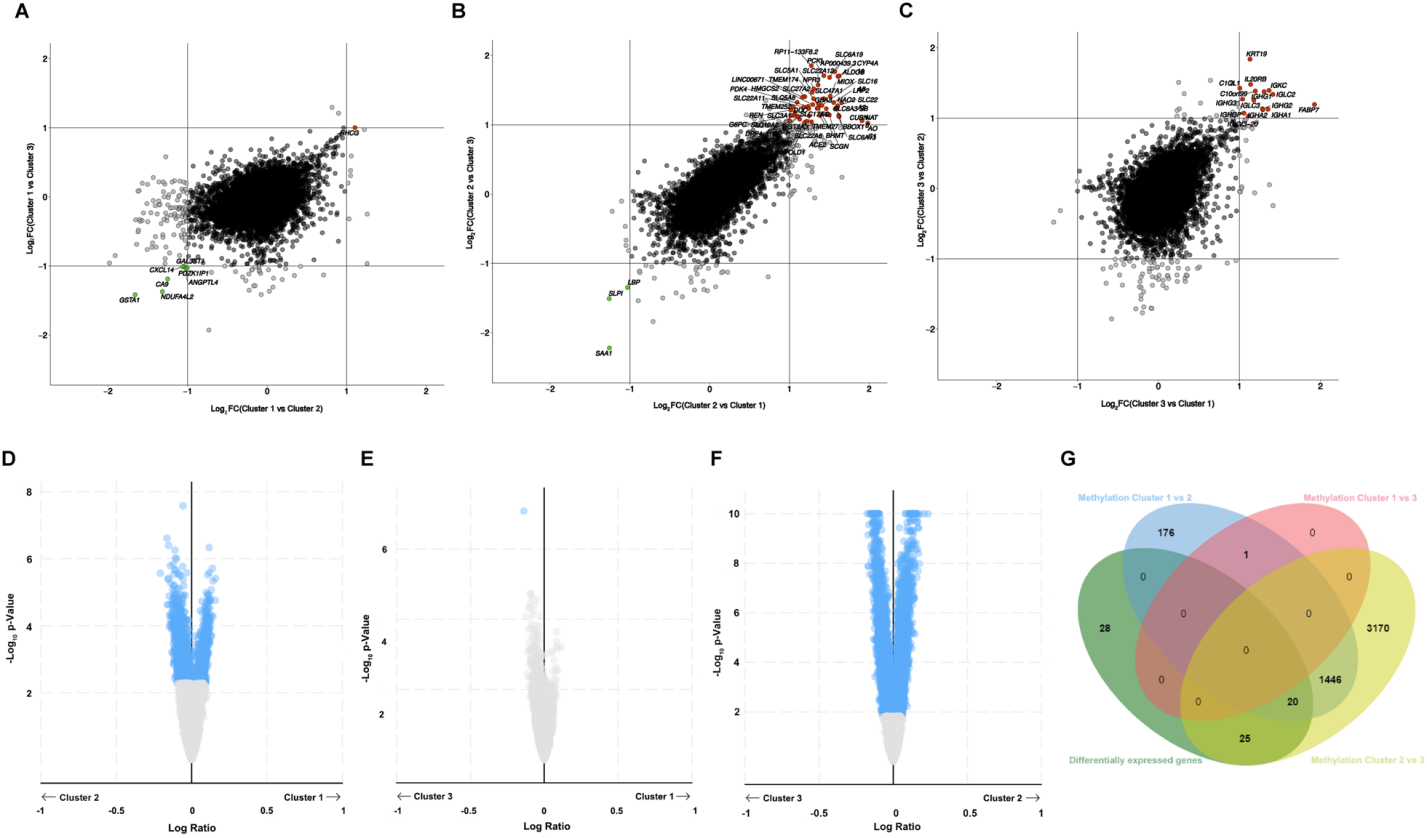
The potential factors for tumor heterogeneity in the three subgroups. **A**–**C** The volcano plots of DEGs between subgroups. **D**–**F** The volcano plots of DNA methylation alterations between subgroups. **G** The Venn diagram showed that there were 78 DEGs, of which only 26 DEGs were not affected by DNA methylation alterations

To explore the potential factors for tumor heterogeneity among CCRCC subgroups, we analyzed whether DEGs among subgroups were associated with DNA methylation alterations, CNVs in the genome and SNPs. Figure 3A–C shows DEGs among subgroups used for the subsequent analysis. Of these, Cluster 1 had one gene with high expression and 7 genes with low expression (Fig. 3A), Cluster 2 had 48 genes with high expression and 3 genes with low expression (Fig. 3B), Cluster 3 had 14 genes with high expression (Fig. 3C). Gene expression levels associated with DNA methylation alterations between subgroups were visualized on volcano maps using the cBioportal (Fig. 3D–F). The data revealed that the significant difference in 1642 genes between Cluster 1 and Cluster 2, one gene between Cluster 1 and Cluster 3, as well as 4661 genes between Cluster 2 and Cluster 3. The Venn diagram revealed that there were 78 DEGs, of which only 26 DEGs (excluding 2 lncRNAs) were not affected by DNA methylation alterations (Fig. 3G).

**Fig. 4:**
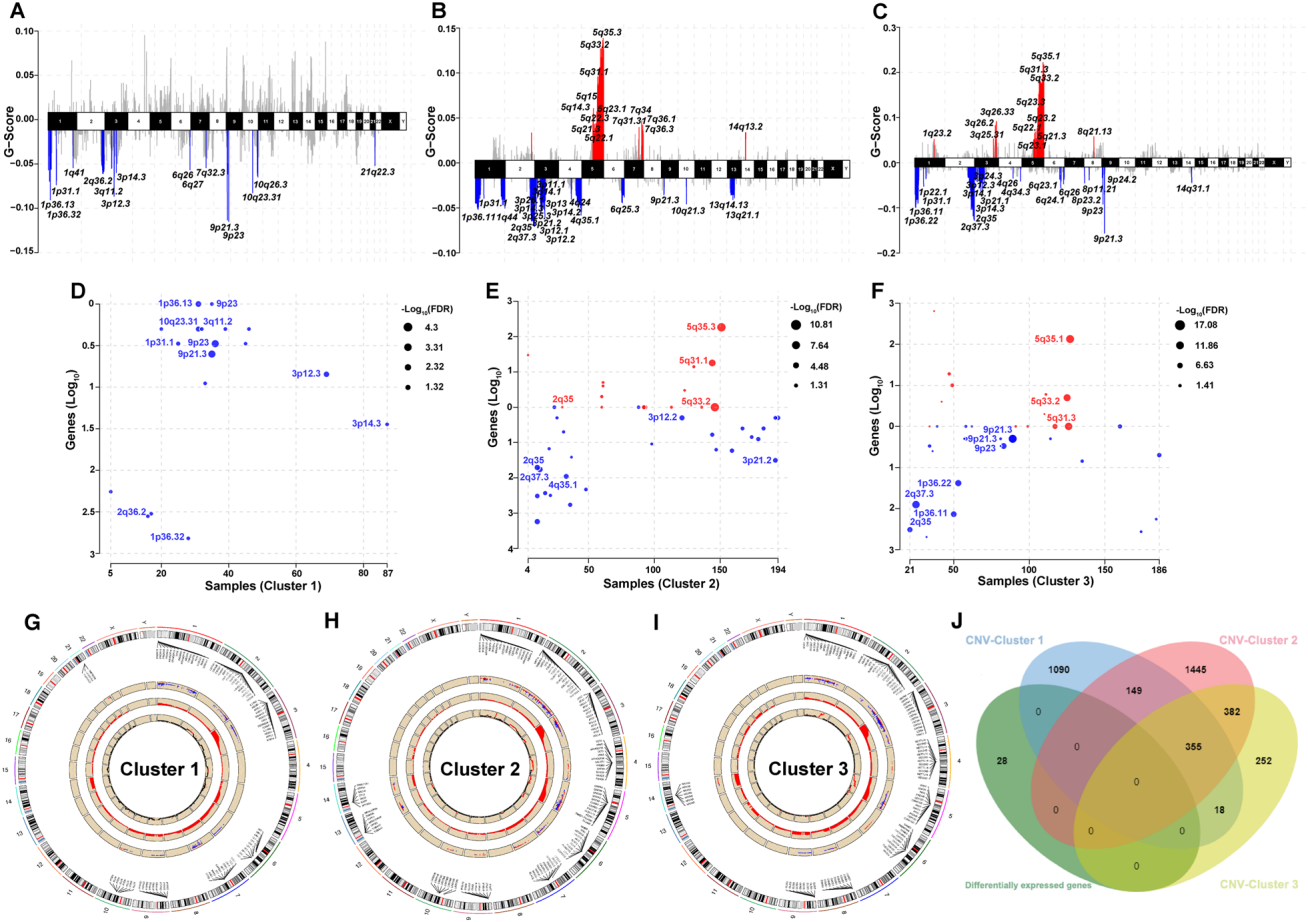
The potential factors for tumor heterogeneity in the three subgroups. **A**–**C** The difference of copy number amplification or deletion in the three subgroups. The red represented the copy number amplification, and the blue represented the copy number deletion. **D**–**F** The alterations of gene expression levels in the three subgroups. The red solid circles represented the copy number amplification, and the blue solid circles represented the copy number deletion. **G**–**I** The specific distribution of copy number amplification and deletion as well as copy number variation-related genes in the three subgroups. **J** The Venn diagram showed that the remaining 26 DEGs also were not affected by CNVs in the genome

CNVs in the genome of CCRCC patients were analyzed using maftools according to the GISTIC algorithm. The results showed that significant copy number amplification or deletion were distributed on different chromosomes (Fig. 4A–C), and the details of copy number amplification and deletion in the genome were presented in Additional file 2: Fig. S2A–F. In addition, the alterations of gene expression levels were analyzed according to the CNVs in the genome. The data revealed that the expression level of 1612 genes in Cluster 1, 2331 genes in Cluster 2 and 1007 genes in Cluster 3 were affected by CNVs (Fig. 4D–F). The specific distribution of copy number amplification and deletion as well as copy number variation-related genes were presented in Fig. 4G–I. The Venn diagram revealed that the remaining 26 DEGs (excluding two lncRNAs) also were not affected by CNVs in the genome (Fig. 4J).

**Fig. 5:**
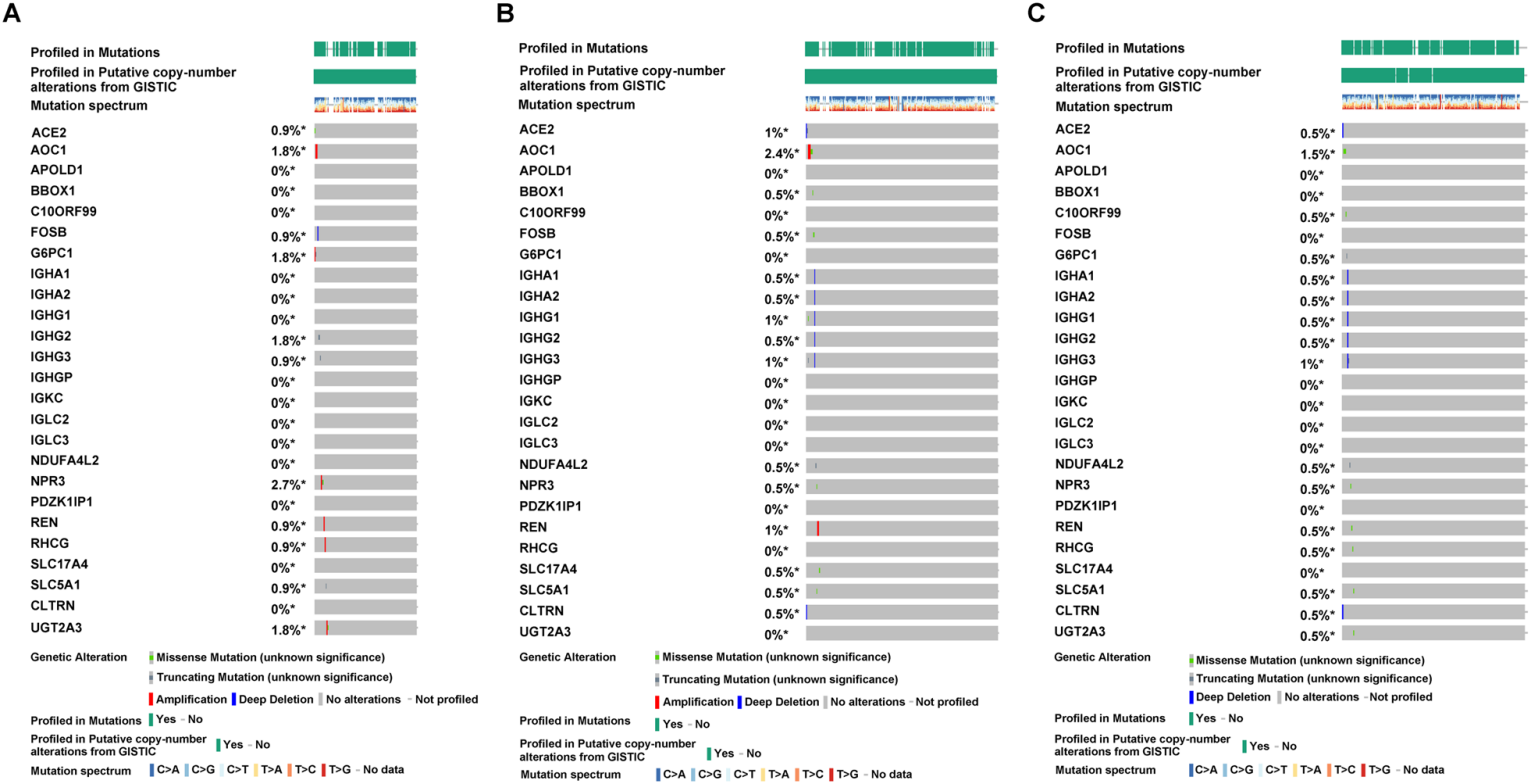
Mutation information of the genes. **A**–**C** The mutation frequencies of 26 DEGs in the 3 subgroups

**Fig. 6:**
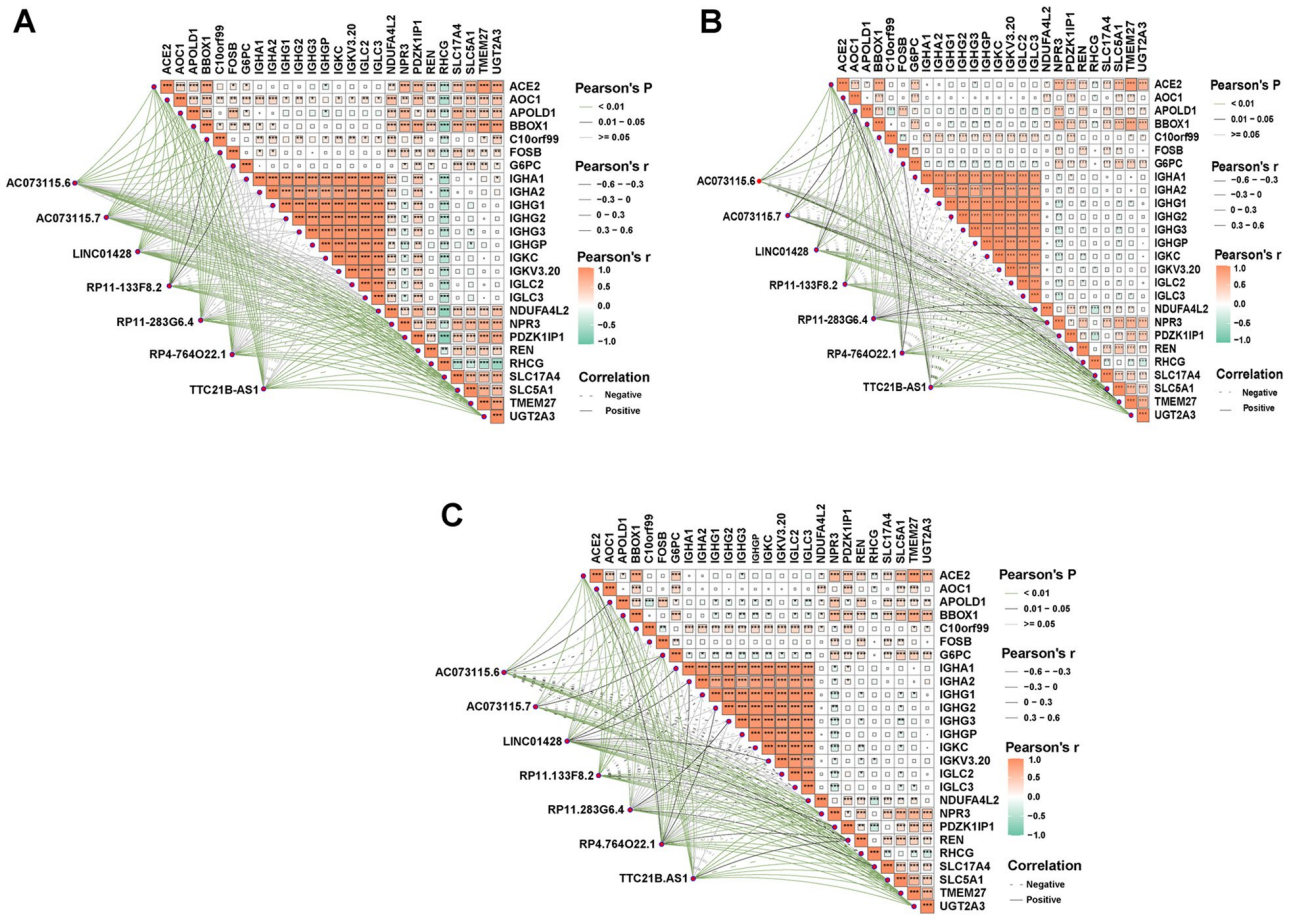
Analysis of correlation. **A**–**C** Correlation between the 26 DEGs in the 3 subgroups and 7 necroptosis-related lncRNAs

Tumorigenesis results from the accumulation of gene mutations [32]. The differences of gene mutations in the three subgroups were analyzed according to the SNPs data. The mutation frequencies of 26 DEGs in each subgroup were presented as waterfall plots (Fig. 5A–C). However, the data revealed that no significant mutations were found in these genes. Finally, the correlation of expression level between the 26 DEGs and 7 necroptosis-related lncRNAs for each subgroup is demonstrated in Fig. 6A–C. The above comprehensive analysis suggested that the 26 DEGs screened may only be associated with 7 necroptosis-related lncRNAs.

### Immune correlation analysis among CCRCC subgroups

**Fig. 7:**
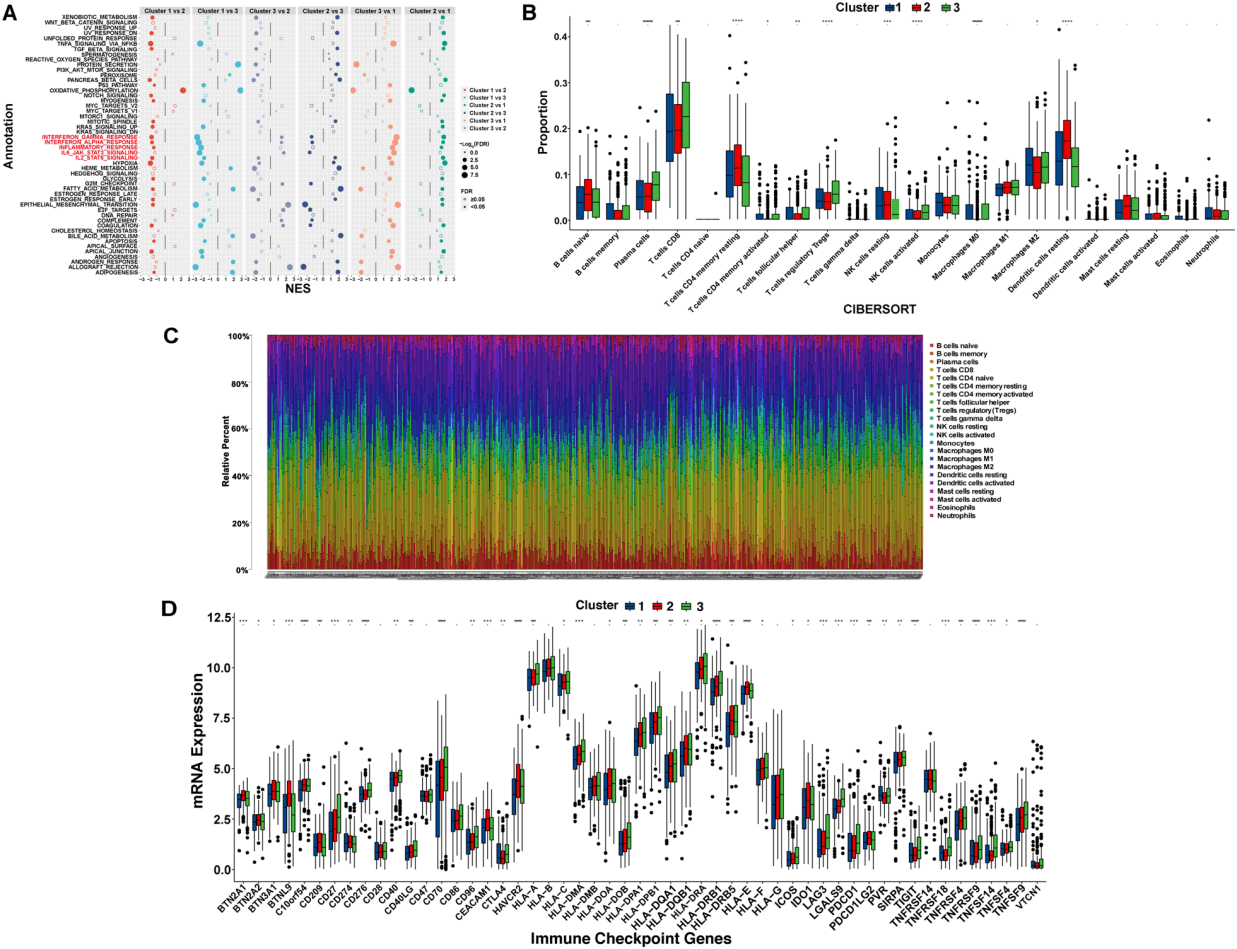
Immunological characteristics. **A** The GSEA of immune-related signaling pathway in the three subgroups. **B** The difference in proportions of TIICs in the three subgroups. **C** The relative percent of TIICs in the enrolled samples. **D** The difference in expression levels of immune checkpoint genes in the three subgroups. *p < 0.05, **p < 0.01, and ***p < 0.001

Based on the different prognosis of CCRCC patients among the three subgroups, we conducted GSEA to explore the underlying differences in biological functions in two subgroups. We found that the INTERFERON_GAMMA_RESPONSE, INTERFERON_ALPHA_RESPONSE, INFLAMMATORY_RESPONSE, IL6_JAK_STAT3_SIGNALING, and IL2_STAT5_SIGNALING were significantly enriched in the Cluster 1, indicating that CCRCC patients in Cluster 1 are closely related to immune-related signaling pathway (Fig. 7A). Subsequently, we further explored the heterogeneity of tumor immune microenvironment among CCRCC subgroups. As shown in Fig. 7B, the differences in the immune cell infiltration among the three subgroups were statistically significant. In addition, the percentage of 22 TIICs in each TCGA-KIRC sample was shown in the bar plot (Fig. 7C). The result revealed that T cells and macrophages were seen to account for the largest components. In addition, we compared the expression level of immune checkpoint genes in the three subgroups and found that almost all the immune checkpoint genes were significantly elevated in the Cluster 2 and 3 (Fig. 7D). These results suggested that Cluster 2 and 3 are more active in immune function and might be more sensitive to immunotherapy.

### Establishment of necroptosis-related lncRNAs predictive risk score

**Fig. 8:**
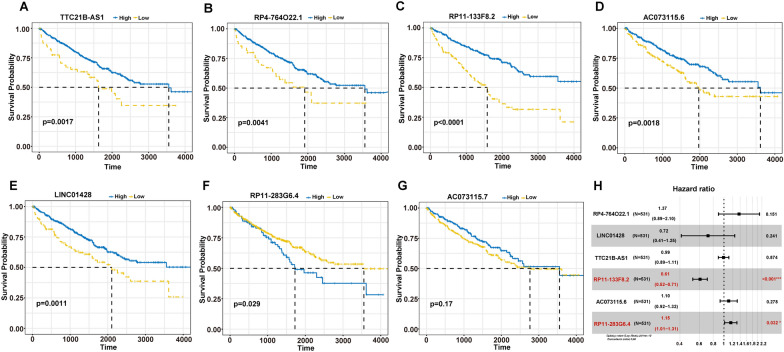
The prognosis value of the seven necroptosis-related lncRNAs. **A** Kaplan–Meier survival curves of high- and low-expression TTC21B-AS1 patients. **B** Kaplan–Meier survival curves of high- and low-expression RP4-764O22.1 patients. **C** Kaplan–Meier survival curves of high- and low-expression RP11-133F8.2 patients. **D** Kaplan–Meier survival curves of high- and low-expression AC073115.6 patients. **E** Kaplan–Meier survival curves of high- and low-expression LINC01428 patients. **F** Kaplan–Meier survival curves of high- and low-expression RP11-283G6.4 patients. **G** Kaplan–Meier survival curves of high- and low-expression AC073115.7 patients. **H** The prognostic necroptosis-related lncRNAs extracted by multivariate Cox regression analysis

To evaluate the prognostic ability of these identified necroptosis-related lncRNAs, we analyzed the relationship between the survival probability and the expression levels of these necroptosis-related lncRNAs. The Kaplan–Meier survival analysis demonstrated that the CCRCC patients with high expression of TTC21B-AS1, RP4-764O22.1, RP11-133F8.2, AC073115.6, and LINC01428 and low expression of RP11-283G6.4 had significantly longer overall survival (Fig. 8A–G). Subsequently, two necroptosis-related lncRNAs (RP11-133F8.2 and RP11-283G6.4) were identified using multivariate Cox regression analysis in TCGA dataset (Fig. 8H). The risk score predictive model was constructed by adding the lncRNAs expression level and relevant coefficient of each lncRNAs as follows: risk score = 【(− 4.970e−01) × RP11-133F8.2 expression】 + 【(1.412e−01) × RP11-283G6.4 expression】.

### Construction of the comprehensive nomogram in TCGA dataset

**Fig. 9:**
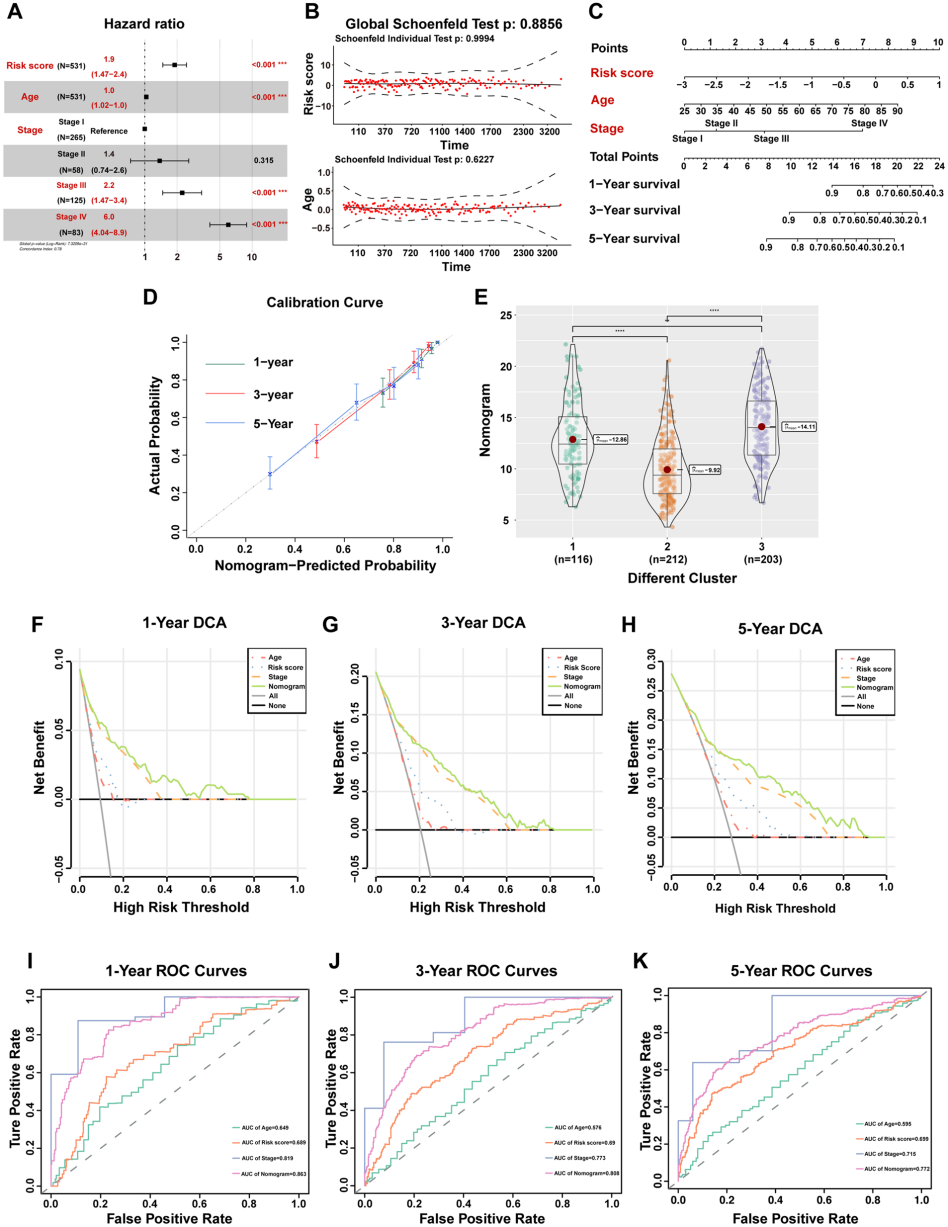
Construction and evaluation of the comprehensive nomogram. **A** Univariate and multivariate Cox analyses of the risk score and clinical factors with OS. **B** The Schoenfeld residual suggested that this model met the equally PH assumption. **C** Comprehensive nomogram, including risk score, age and stage, was established to predict 1-, 3-, and 5-year OS probability in CCRCC. **D** The calibration curves of 1, 3, and 5 years showed more appropriate calibration ability. **E** The different score of nomogram in the three subgroups. **F**–**H** The DCA curves showed a comparable net benefit if the threshold probability for a patient or a doctor was within a range from 0 to 0.80 during 1, 3, and 5 years. **I**–**K** The time-dependent ROC curve analysis for the nomogram and single indicator during 1, 3, and 5 years, respectively

Based on our previous results, we constructed a new nomogram using risk score combined with clinical indicators for preoperatively evaluation of patients’ survival and therapy response. Univariate Cox regression followed by multivariate Cox regression was used to identify the most significant independent risk/protective factors. The forest plot showed the risk score, age, stage III and stage IV are independent risk factors (Fig. 9A). The Schoenfeld residual test showed that all of the variables met equally proportional hazards (PH) assumption (Fig. 9B). Considering all the identified significant predictive factors, we construct a comprehensive nomogram including risk score, age and stage to predict the 1-, 3-, and 5-year OS rates of CCRCC patients (Fig. 9C). The calibration curves displayed suitable calibration efficiency indicating a good consistency between the actual OS rates and predicted survival rates at 1, 3, and 5 years (Fig. 9D). Subsequently, we compared the scores of the nomogram among the three subgroups and found that Cluster 2 had the lowest score and Cluster 3 had the highest score. The result was consistent with the previous survival analysis among the three subgroups, indicating that the nomogram may be used to guide clinical prognostic analysis (Fig. 9E). The DCA was used to evaluate the clinical application of nomogram and the net benefits of different prediction models at different threshold probabilities. As shown in Fig. 9F–H, the new nomogram showed better net benefit than age, stage and risk score. Moreover, the time-dependent ROC curve verified that prediction performance of the nomogram was better compared to the other index (Fig. 9I–K).

### Analysis of qRT-PCR

**Fig. 10:**
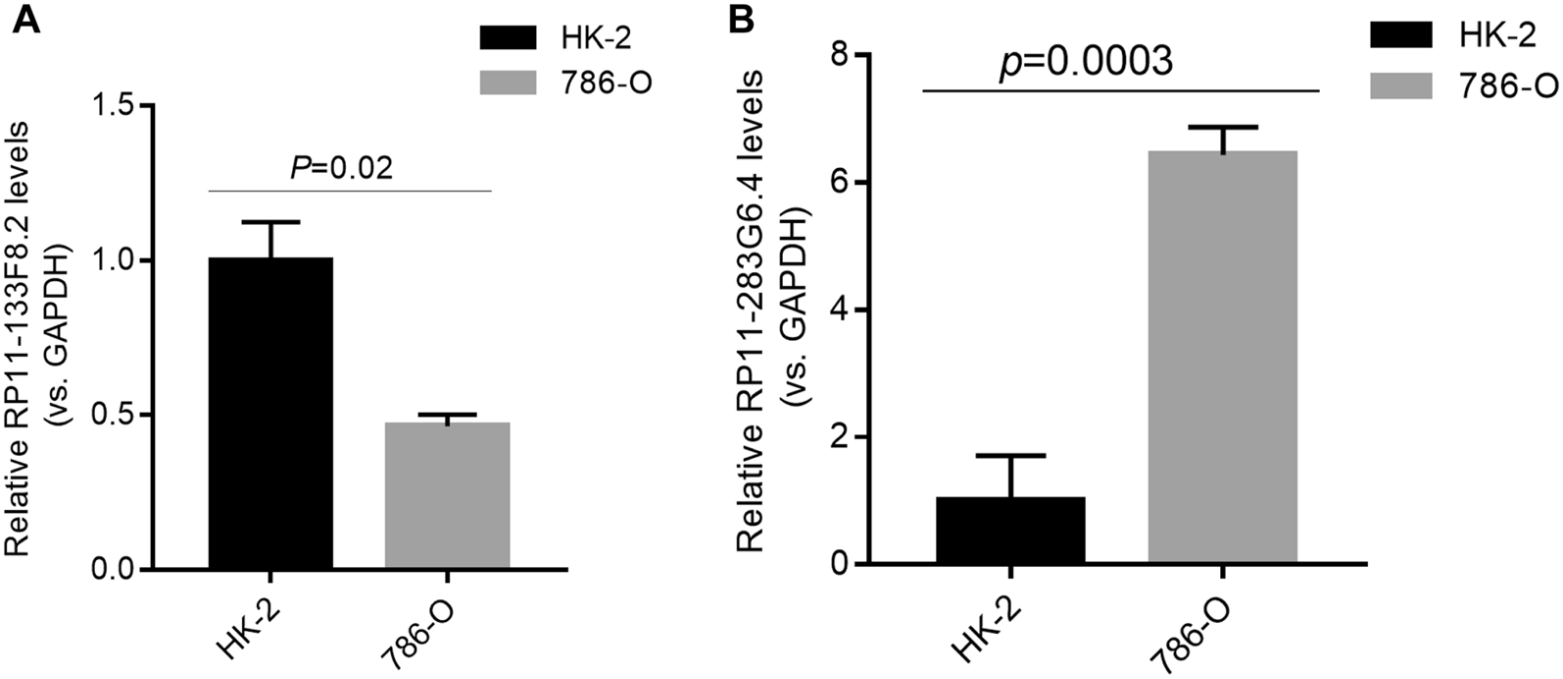
The qRT-PCR results of the two necroptosis-related lncRNAs relative expression levels. **A** The qRT-PCR showed that the relative expression levels of RP11-133F8.2 in two lineage cells (HK-2 and 786-O). **B** The qRT-PCR showed that the relative expression levels of RP11-283G6.4 in two lineage cells (HK-2 and 786-O)

Two necroptosis-related lncRNAs (RP11-133F8.2, RP11-283G6.4) were selected for further analysis. The two lncRNAs were tested in HK-2 and 786-O cells. As shown in Fig. 10A, B, the two lncRNAs are differentially expressed between tumor and normal renal cells. The expression of RP11-133F8.2 was reduced in CCRCC cells, and the expression of RP11-283G6.4 was elevated in CCRCC cells compared with renal proximal convoluted tubule cells. This means that the experimental results also confirmed the reliability of the risk model.

## Discussion

RCC, a urological malignant tumor, represents approximately 90% of all malignancies of the kidney [3]. Due to the aggressive nature of RCC, 20–30% of patients have synchronous metastases at the time of initial diagnosis, and 20–40% of patients develop metachronous metastases after nephrectomy [33]. Although a combination of immune checkpoint inhibitors or a combination of immune checkpoint inhibitor and tyrosine kinase inhibitors has achieved significant therapeutic improvement in the treatment of metastatic RCC, the 5-year survival rate of these patients remains poor [5, 6, 34]. In our study, two necroptosis-related lncRNAs, including RP11-133F8.2 and RP11-283G6.4, could be used to predict the survival outcome of patients with CCRCC, and further study of these lncRNAs may be helpful for individualized treatment of these patients.

LncRNAs are a class of transcribed non-coding RNAs with a length of more than 200 nucleosides, which affects gene expression of protein coding genes in several ways [35]. Many lncRNAs play an important role in the initiation, evolution and progression of RCC, as well as in the development and spread of metastases [35]. Chen found that lncRNAs SNHG12 promoted RCC proliferation, migration invasion and sunitinib resistance via CDCA3 in vitro and increased tumor growth in vivo [36]. Zhang confirmed that lncRNAs DARS-AS1 promotes the progression of CCRCC by sequestering miR-194-5p to up-regulate DARS [37]. Dong revealed that lncRNAs TUG1 promotes cell proliferation and inhibits cell apoptosis and autophagy in CCRCC via miR-31-5p/FLOT1 axis [38]. In recent years, many studies have highlighted the critical role of lncRNAs in CCRCC. However, the relationship between them is still unclear.

Necroptosis is a new programmed form of necrotic cell death that shares mechanistic similarities with apoptosis and morphological similarities with necrosis [39]. Recent studies have revealed a significant role of necroptosis in tumorigenesis and metastasis and implicated the potential of targeting necroptosis as a novel cancer therapy [40]. However, the mechanism of necroptosis in cancer is still unclear. In our study, a preliminary classification of molecular subgroups was performed based on the expression of these identified necroptosis-related lncRNAs and survival was further analyzed in these classified three subgroups. Furthermore, we systematically studied the correlation between the tumor heterogeneity in the three subgroups and necroptosis-related lncRNAs and look forward to using these results to contribute to the mechanism research of necroptosis-related lncRNAs in CCRCC.

In this study, we first carried out differential expression analysis and 138 lncRNAs were significantly differentially expressed between CCRCC and normal groups. WGCNA was performed to analyze the expression values of 138 lncRNAs and 7 necroptosis-related lncRNA were screened out for the subsequent analysis. Then, NMF consensus clustering analysis was used to divide these identified lncRNAs into three subgroups. In addition, survival analysis showed that Cluster 2 had a better survival probability than Cluster 1 and Cluster 3. To explore the potential factors for tumor heterogeneity in three subgroups, we revealed that only 26 DEGs were not affected by DNA methylation alterations, CNVs and SNPs. On the contrary, these DEGs were associated with the seven necroptosis-related lncRNA. Subsequently, survival analysis demonstrated that the CCRCC patients with high expression of TTC21B-AS1, RP4-764O22.1, RP11-133F8.2, AC073115.6, and LINC01428 and low expression of RP11-283G6.4 had significantly longer overall survival. The risk score prognostic model was constructed by multivariate Cox regression analysis, and two necroptosis-related lncRNAs were used to establish the risk model. Based on our previous results, we constructed a new nomogram using risk score combined with clinical indicators for preoperatively evaluation of patients’ survival and therapy response. The scores of the nomogram among the three subgroups were compared and the results showed that Cluster 2 had the lowest score and Cluster 3 had the highest score. This was consistent with the previous survival analysis among the three subgroups, indicating that the nomogram may be used to guide clinical prognostic analysis. In addition, we also analyzed the level of immune cell infiltration and immune checkpoint genes among the three subgroups. These results may provide significant evidence for new targets of immunotherapy in the future.

An increasing number of studies have shown that necroptosis-related lncRNAs are associated with the occurrence and progression of malignant tumors, but the relevant research about CCRCC in this field remains unclear. In this study, we first constructed gene co-expression networks by WGCNA to screen out seven necroptosis-related lncRNAs for the subsequent analysis. Second, we comprehensively analyzed the potential reasons for the tumor heterogeneity among CCRCC subgroups for the first time, providing new insights for further research into the molecular mechanisms of necroptosis-related lncRNAs. However, our study has some limitations. First, all data for this study were downloaded from TCGA, which may lead to bias in the relevant analysis. Second, we did not perform relevant experiments to validate the differences in the levels of molecular transcription and expression, which may reduce its credibility. Third, we lacked follow-up information on CCRCC patients to demonstrate the clinical value of our prognostic model.

## Conclusions

In this study, we comprehensively evaluated the value of necroptosis-related lncRNAs in predicting survival, the potential factors of tumor heterogeneity, and the role of the tumor immune microenvironment. A novel risk model was constructed based on two necroptosis-related lncRNAs, including RP11-133F8.2 and RP11-283G6.4, which could be used to predict the survival outcome of patients with CCRCC. These findings contributed to understand the function of necroptosis-related lncRNAs in CCRCC and provided new insights of prognosis assessment and optimal therapeutic strategy for CCRCC.

## Supplementary Information

**Additional file 1: Figure S1.** Graphs of scale independence, mean connectivity and scale-free topology.

**Additional file 2: Figure S2.** The details of copy number amplification and deletion in the genome in the three subgroups.

**Additional file 3: Table S1.** Identification of differentially expressed genes.

**Additional file 4: Table S2.** Necroptosis-related genes.

**Additional file 5: Table S3.** Distribution of lncrnas per module.

**Additional file 6: Table S4.** Survival information of enrolled CCRCC patients.

## Data Availability

Public data used in this work can be acquired from the TCGA Research Network portal (https://portal.gdc.cancer.gov/). The raw experimental data and analysis codes supporting the conclusions of this article will be made available by the corresponding author.

## Abbreviations

RCC: Renal cell carcinoma
CCRCC: Clear cell renal cell carcinoma
LncRNAs: Long non-coding RNAs
RIPK1: Receptor-interacting protein kinase 1
RIPK3: Receptor-interacting protein kinase 3
MLKL: Mixed lineage kinase domain-like pseudokinase
DAMPs: Damage-associated molecular patterns
TIICs: Tumor-infiltrating immune cells
TCGA: The Cancer Genome Atlas
WGCNA: Weighted gene co-expression network analysis
NMF: Non-negative matrix factorization
PCA: Principal component analysis
FGA: Fraction genome altered
TMB: Tumor mutational burden
CNVs: Copy number variations
SNPs: Single nucleotide polymorphisms
DCA: Decision curve analysis
ROC: Receiver-operating characteristics
DEGs: Differentially expressed genes
GSEA: Gene set enrichment analyses
qRT-PCR: Quantitative real-time polymerase chain reaction
