# Supplementary figures and images for: Comprehensive analysis of necroptosis-related lncRNA signature with potential implications in tumor heterogeneity and prediction of prognosis in clear cell renal cell carcinoma
Source: Eur J Med Res. 2023 Jul 14;28:236. doi: 10.1186/s40001-023-01194-4 (PMC10347828; doi:10.1186/s40001-023-01194-4)

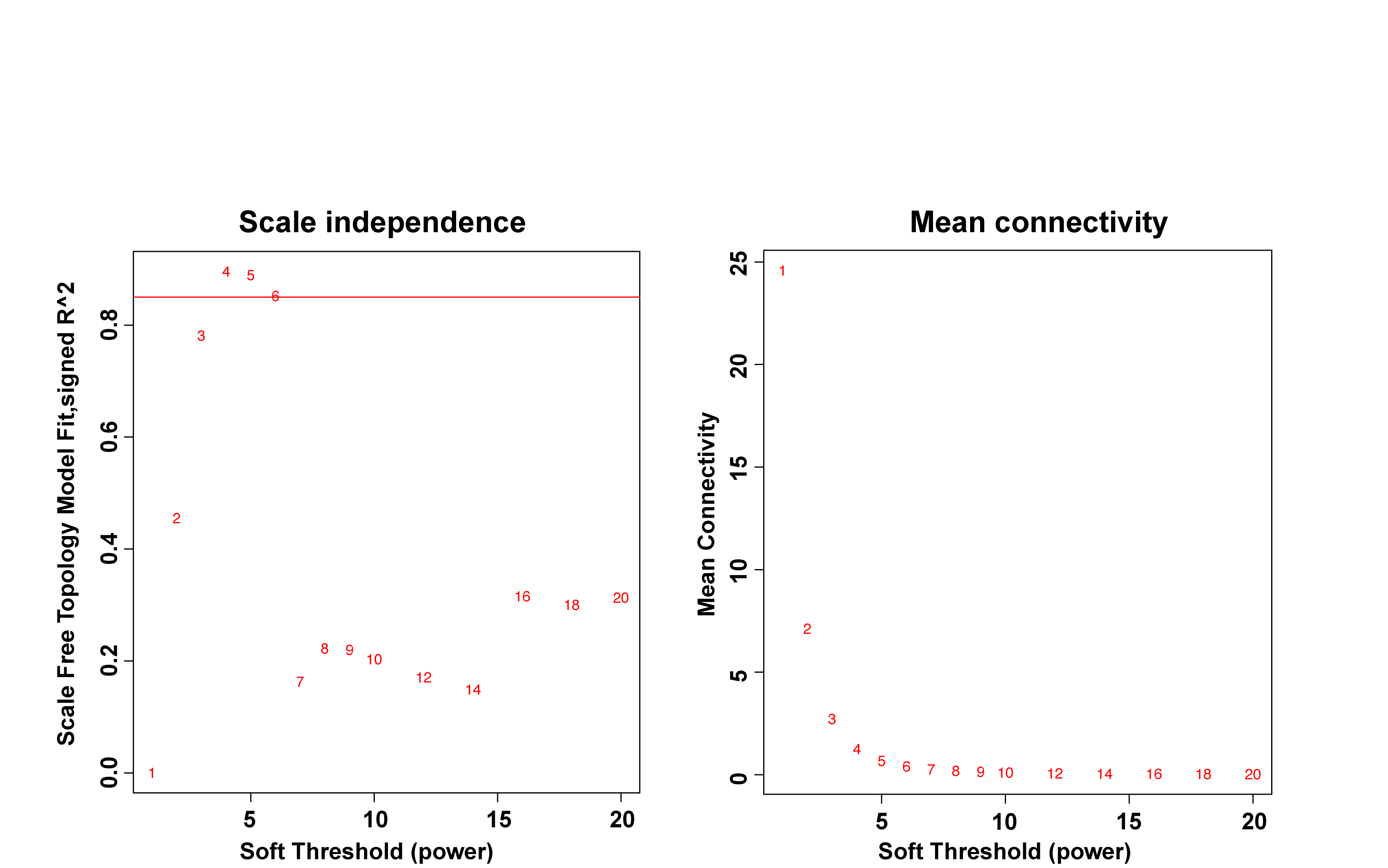

Supplement: Supplementary file 1 — Additional file 1: Figure S1. Graphs of scale independence, mean connectivity and scale-free topology. [file 40001_2023_1194_MOESM1_ESM.tif]

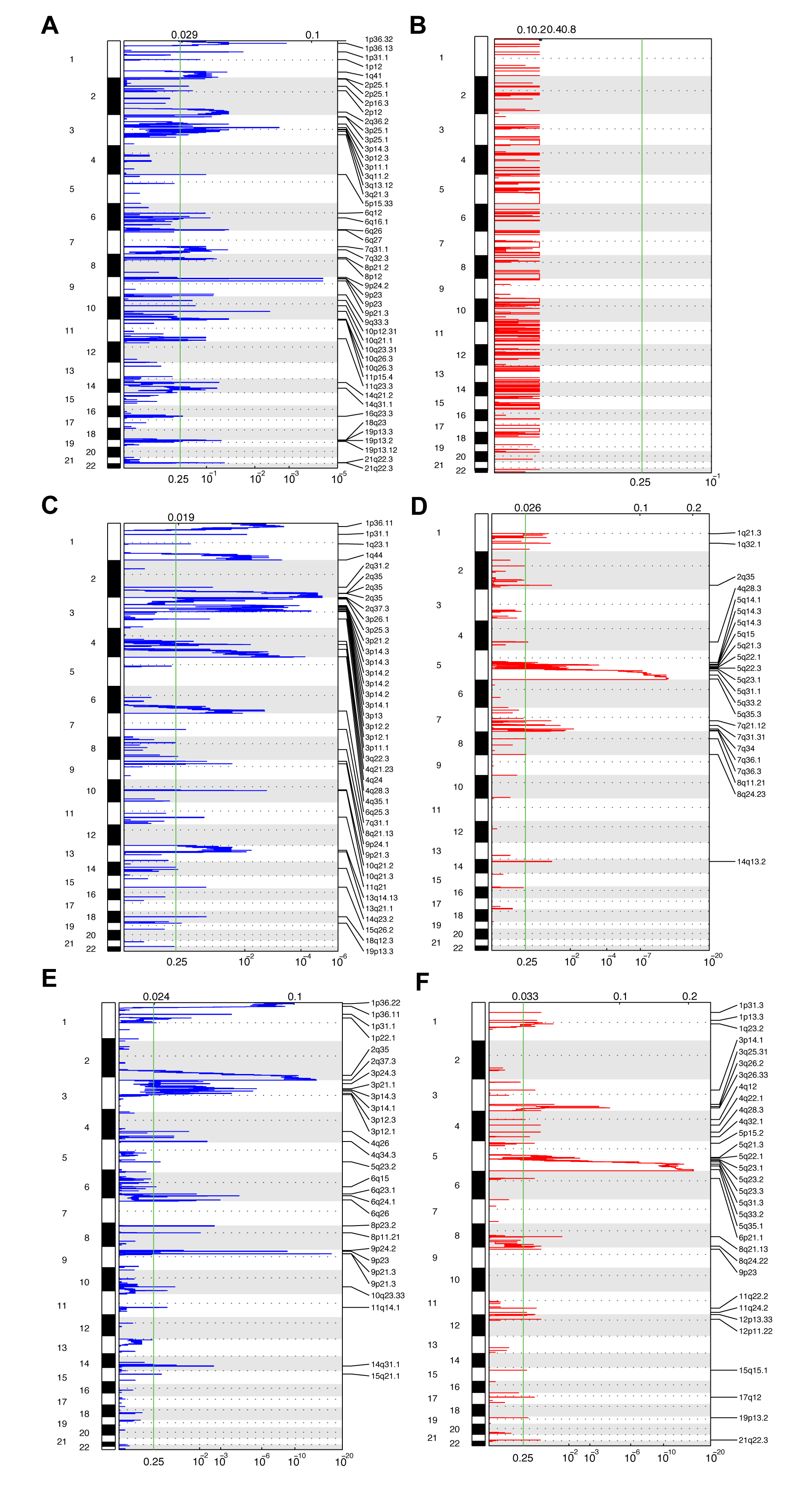

Supplement: Supplementary file 2 — Additional file 2: Figure S2. The details of copy number amplification and deletion in the genome in the three subgroups. [file 40001_2023_1194_MOESM2_ESM.tif]
